# A case of pityriasis rubra pilaris secondary to ponatinib

**DOI:** 10.1177/2050313X241311341

**Published:** 2025-01-08

**Authors:** Ariana Nateghi, Florence Lagacé-Thomassin, Julie Desrochers

**Affiliations:** 1 Department of Dermatology, Sherbrooke University, Sherbrooke, QC, Canada; 2 Departement of Dermatology, Laval University, Quebec, QC, Canada; 3 Departement of Dermatology, Charles-Le Moyne Hospital, Longueuil, QC, Canada

**Keywords:** Case report, ponatinib, leukemia, Philadelphia chromosome-positive, pityriasis rubra pilaris, tyrosine kinase inhibitor

## Abstract

Ponatinib, a tyrosine kinase inhibitor used for chronic myeloid leukemia and acute lymphoblastic leukemia, can cause rare cutaneous side effects. In this case, a 63-year-old woman developed a pityriasis rubra pilaris-like eruption 1 month after starting the drug. The skin reaction improved with dose reduction and recurred more mildly at a lower dose. Symptomatic relief was achieved with topical tretinoin, triamcinolone, and emollients. This case underscores the importance of managing dose-dependent skin reactions while maintaining cancer therapy.

Ponatinib is an oral tyrosine kinase inhibitor (TKI) used primarily for treating refractory chronic myeloid leukemia (CML) and Philadelphia chromosome-positive acute lymphoblastic leukemia (ALL). Although generally well tolerated, ponatinib is known for causing a thrombocytopenia, rash, abdominal pain, and dry skin.^1^ To date, the literature showed that 13 cases of these kinds of side effects on the skin were reported.

This case presents a 63-year-old female patient who visited the dermatology clinic in January 2023 with a pruritic skin eruption that began 1 month after starting ponatinib in April 2022. Her history includes diffuse large B-cell lymphoma treated with chemotherapy in 2017, followed by a bone marrow transplant in 2019. In December 2020, she was diagnosed with B-cell ALL, initially treated with vincristine, dexamethasone, and imatinib, later transitioning to dasatinib. Due to a lack of response, dasatinib was discontinued, and ponatinib was started. By January 2023, her ponatinib dose was reduced to 30 mg to manage the skin eruption.

On examination, the patient displayed large, well-demarcated, scaly orange-red erythematous plaques with follicular hyperkeratotic papules (Figure 1). These plaques were located mainly on the neck, anterior thorax, axillary folds, hips, and buttocks, with some isolated plaques on the limbs. There was no evidence of palmoplantar keratoderma, and the systemic review was unremarkable. A clinical diagnosis of ponatinib-induced pityriasis rubra pilaris-like eruption was made. Although the patient reported mild pruritus, the eruption was considered manageable, allowing the continuation of her cancer treatment. A topical treatment regimen consisting of 0.01% tretinoin cream, 0.1% triamcinolone cream, and an emollient was prescribed. Notably, by the time of the dermatology evaluation, her skin condition had already improved, which coincided with the reduction of the ponatinib dose from 45 to 30 mg. A few months later, the patient temporarily discontinued ponatinib due to pancreatitis after which the eruption resolved spontaneously. Upon resuming treatment at 15 mg, the eruption recurred, though it was less intense.

**Figure 1.**
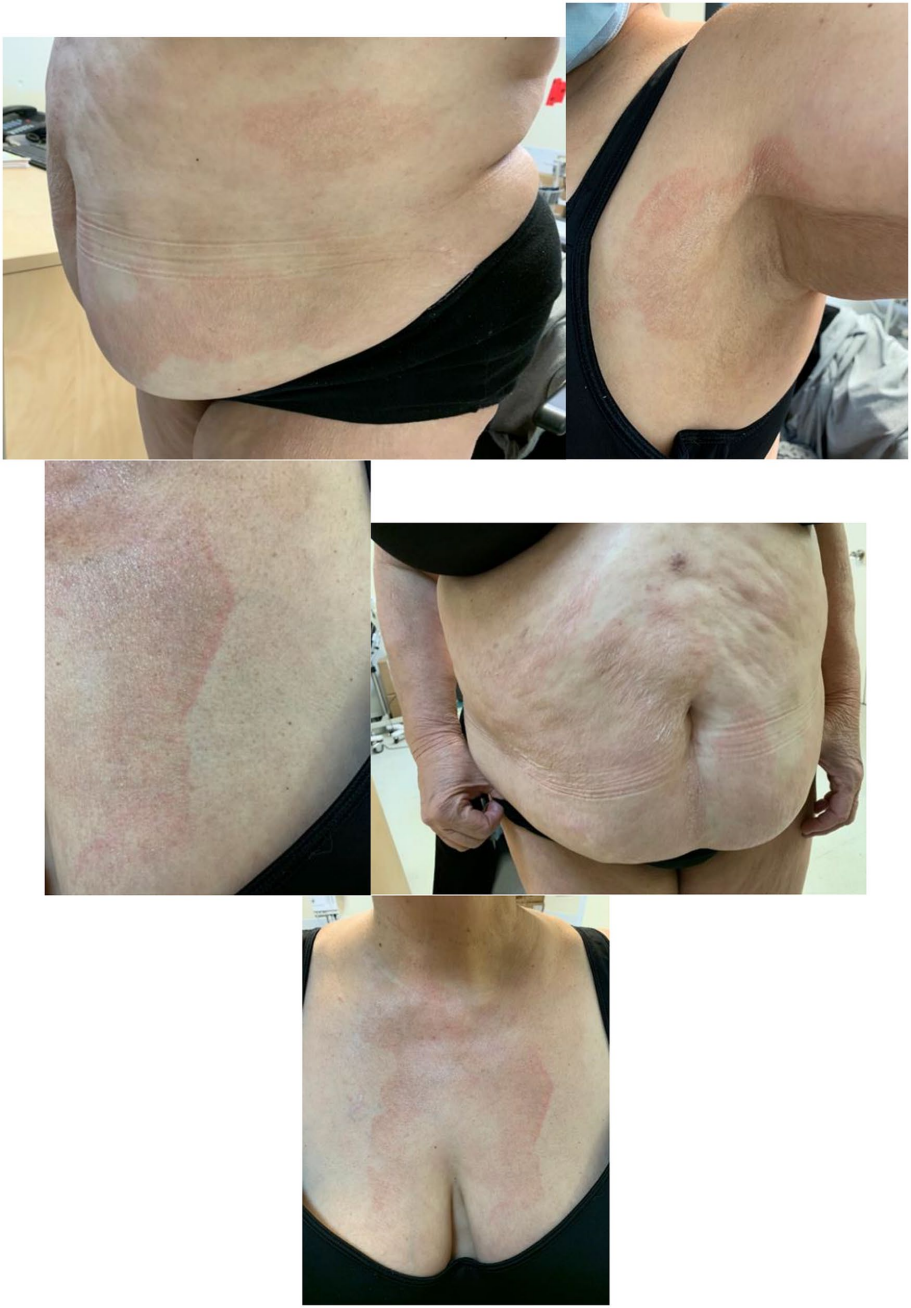
Well-demarcated, scaly orange-red erythematous plaques with follicular hyperkeratotic papules.

Ponatinib is known for causing several adverse effects, with cutaneous reactions being among the most common. According to the PACE trial, 47% patients experienced skin rashes, and 42% reported dry skin.^1,2^ However, cases of ponatinib-induced pityriasis rubra pilaris-like eruptions are fairly rare. This case demonstrated a clear dose–dependent relationship between ponatinib and the skin eruption, as dose reduction improved symptoms, and a lower dose resulted in a milder recurrence. Management of ponatinib-induced skin reactions typically includes the use of topical or systemic retinoids and corticosteroids. In this case, a combination of tretinoin and triamcinolone, alongside emollient use, provided symptomatic relief, allowing the patient to continue therapy.^3,4^ Reports in the literature support this approach, highlighting the efficacy of similar strategies in mitigating dermatologic complications without interrupting cancer treatment.

As TKIs like ponatinib expand, recognizing and managing rare side effects, such as pityriasis rubra pilaris-like eruptions, becomes crucial. With prompt diagnosis and management, patients can continue on life-saving therapies while minimizing complications. The successful resolution of this case highlights the importance of a tailored approach, balancing oncologic efficacy with patient’s quality of life. Early intervention ensures treatment continuation, optimizing the outcomes for patients with complex malignancies.
